# The prevalence of anxiety symptoms in infertile women: a systematic review and meta-analysis

**DOI:** 10.1186/s40738-020-00076-1

**Published:** 2020-04-15

**Authors:** Zahra Kiani, Masoumeh Simbar, Sepideh Hajian, Farid Zayeri, Maryam Shahidi, Marzieh Saei Ghare Naz, Vida Ghasemi

**Affiliations:** 1 Student Research Committee, Department of Midwifery and Reproductive Health, School of Nursing and Midwifery, Shahid Beheshti University of Medical Sciences, Tehran, Iran; 2 Midwifery and Reproductive Health Research Center, School of Nursing and Midwifery, Shahid Beheshti University of Medical Sciences, Tehran, Iran; 3 Proteomics Research Center and Department of Biostatistics, Faculty of Paramedical Sciences, Shahid Beheshti University of Medical Sciences, Tehran, Iran; 4 Department of Biochemistry and Biophysics, Mazandaran University of Medical Sciences, Sari, Iran; 5 Hazrat-e Maryam Fertility Center, Sari, Iran

**Keywords:** Infertility, Women, Anxiety, Meta-analysis, Prevalence

## Abstract

**Background:**

Infertile women are exposed more frequently to anxiety risk than are infertile men, thereby adversely affecting the procedures with which they are treated and the quality of their lives. Yet, this problem is often disregarded. This study accordingly determined the prevalence of anxiety symptoms among infertile women.

**Methods:**

All Persian and English studies published from the early 2000s to May 2019 were searched in international (i.e., PubMed, the Cochrane Library, Web of Science, Scopus, Embase, and PsycINFO) and national (i.e., SID, Magiran) databases as well as through Google Scholar. After the titles and abstracts of the articles were reviewed, their quality was evaluated, and relevant works for examination were selected in consideration of established inclusion and exclusion criteria. The risk of biases of individual studies according to Newcastle - Ottawa Scale was assessed. The heterogeneity of the studies was assessed using the I^2^ statistic, and indicators of publication bias were ascertained using Egger’s test. Stata (version 14) was employed in analyzing the findings.

**Results:**

Thirteen studies having a collective sample size of 5055 infertile women were subjected to meta-analysis, with study heterogeneity incorporated into a random effects model. The findings indicated that 36% of the infertile women involved in the evaluated studies self-reported their experience with anxiety. The pooled prevalence of the condition among the subjects was 36.17% [95% confidence interval (CI): 22.47–49.87]. The pooled prevalence levels in low- and middle-income countries and high-income countries were 54.24% (95% CI: 31.86–78.62) and 25.05% (95% CI: 15.76–34.34), respectively. The results revealed no evidence of publication bias (P _Egger’s test_ = 0.406).

**Conclusion:**

Considering the prevalence of anxiety in infertile women and its effects on health processes and quality of life, this problem requires serious consideration and planning for effective intervention, especially in low- and middle-income nations.

## Introduction

Infertility is defined by the World Health Organization (WHO) as the inability to conceive after 1 year (or longer) of unprotected intercourse [1]. Its prevalence has increased by 50% since the past decade, with rates ranging from 9 to 18% in different parts of the world [2]. A systematic analysis of infertility incidence revealed that 1.9% of women experience primary infertility and 10.5% suffer from secondary infertility [3]. Infertility among couples might originate from male factors (35%), tubal and pelvic pathology (35%), ovulatory dysfunction (15%), unexplained infertility (10%), and unusual problems (5%) [4]. The WHO regards infertility as an important reproductive health problem that causes emotional, psychological, and social disorders [1], but the invisibility of this condition means that it has stimulated minimal concern [5] and has thus prevented afflicted individuals from exercising control over their lives [6].

Having a child is of considerable importance to women, and when they are unable to conceive, they are often subjected to strong pressure from family members and relatives [7]. Infertility threatens women’s families and social statuses and, in some societies, may drive husbands to seek a divorce or re-marry [8]. In many communities, infertility is considered a feminine condition, thereby rendering the condition a general stigma with devastating consequences. The actual and potential problems (emotional, psychological, and social disorders) stemming from infertility cause anxiety in women [7, 9] Part of that is due to the lack of infertility services in the primary health care system [10]. Interventions are offered mostly at high costs in private clinics, thus restricting the availability of these services, especially in low- and middle-income countries [11, 12] Further pressure arises because women, especially in developing and low-income countries, are typically the recipients of tests and treatments, they are mostly unemployed, and men shoulder expenses related to therapies [10, 11]. The majority of infertility women are more likely to expose to negative effects on quality of life than men—a phenomenon that increases anxiety among affected female populations [13–15].

Most studies show that marital quality of infertile couples decreases and this can be related to the couple’s anxiety symptoms [11, 12]. Few studies uncovered that the quality of marital relations between infertile couples is better than that between their fertile counterparts as the disease drives couples to be closer and reduces the associated apprehension [16, 17]. Nevertheless, infertility and its treatment continue to be important factors for anxiety among women because of the long-term nature of intervention and the unpredictability of success. The administration of medication and referring to a doctor can lead to anxiety [18]. Apprehension can likewise be due to the difficulties associated with drug use and therapeutic measures, which in turn, affect the outcomes of infertility treatment [19, 20].

Global reports indicated differences in the prevalence of anxiety in infertile couples [21] as infertile women experience a higher level of anxiety than that experienced by infertile men, and the inability to conceive affects anxiety prevalence among the former [22]. The diversity in findings is reflected, for example, in the works of Alosaimi et al. [23], Maroufizadeh et al. [24], and Volgsten et al. [25], who found that anxiety prevalence among infertile women in Saudi Arabia, Iran, and Sweden reached rates of 21.8, 58.1, and 14.8%, respectively. Despite the value presented by these studies, however, no meta-analytic research has been devoted to specifically outlining anxiety prevalence with regard to female factors. One of the goals of meta-analysis was to provide accurate and valid information on the basis of a large sample derived from the combination of studies; the insights obtained offer data that can help physicians and service providers develop interventions and treatments [26]. Correspondingly, this study conducted a systematic review and meta-analysis to probe into the prevalence of anxiety symptoms in infertile women, with exclusive focus directed toward factors relevant to female populations.

## Methods

### Search strategies

The preferred reporting items for systematic reviews and meta-analyses (PRISMA) [27] was used to identify and articulate the problem explored in this work, collect and analyze data, interpret the findings, and draw conclusions. We conducted a comprehensive search in international databases, namely, PubMed, the Cochrane Library, Web of Science, Scopus, PsycINFO, and Embase, as well as national databases, namely, SID and Magiran. A Google Scholar search was also performed. Relevant Persian and English articles published from the early 2000s to May 2019 were searched and extracted by two independent researchers on the basis of keywords (i.e., “anxiety,” “anxiety disorders,” “infertility,” “prevalence,” “epidemiology”) that were combined using the AND and OR operators to ensure a comprehensive and complete search process. The details of the search strategy are in Additional file 1.

### Inclusion and exclusion criteria

The inclusion criteria were as follows: cross-sectional studies, only female factor for infertility not male factor or both or unknown factors, female factor-based reports on anxiety prevalence in infertile women, Studies with valid measurement tool using a validated cutoff score or clinical interview, studies published from 2000 to 2019, studies that involved a minimum sample size of 30, and studies that had samples being of reproductive age (15–49 years), unable to achieve pregnancy following at least 1 year of unprotected intercourse, suffering from primary or secondary infertility, and lacking non-chronic diseases.

The exclusion criteria were lack of full access to articles, studies with irrelevant reports, similar studies, other types of research (review, meta-analysis, interventional, cohort, and case-control studies, etc.), studies on infertile men or couples wherein infertility factors were not distinguished (i.e., female factors, male determinants, both factors, or unknown factors), and studies featuring pregnancy in women initially diagnosed as infertile.

### Data extraction

Relevant Persian and English articles published from the early 2000s to 31 May 2019 were searched. The keyword search first yielded 282,760 articles. After the exclusion of identical studies, the titles and abstracts of the remaining articles were reviewed. When necessary, the main texts were examined. All the steps were independently evaluated by two reviewers, and a third reviewer was involved in case of disagreement. The required data, including the names of authors, years of publication, countries where the studies were conducted, sample sizes, anxiety prevalence in infertile women, types of tools used to measure anxiety (standard instruments or.

interviews for diagnostic purposes to determine anxiety), mean ages of women, and durations of infertility, were obtained.

### Quality evaluation

Wells et al. [28], developed a valid and reliable checklist called the Newcastle–Ottawa Scale (NOS) to assess the quality of non-randomized studies through meta-analyses. The quality of the present research was evaluated using the modified version of the NOS by Zhang et al. [29], which addresses five domains: the representativeness of a sample (Population contained a mixture of specialties at multiple sites or a single specialty at a single site), sample size (200 and greater than 200 participants or less than 200), non-respondents (Comparability between respondent and non-respondent characteristics was established, and the response rate was satisfactory), the ascertainment of anxiety (Validated measurement tool using a validated cutoff score or clinical interview), and the quality of descriptive statistics reporting Reported descriptive statistics to describe the population (e.g., age, sex) with proper measures of anxiety (e.g., standard deviation, standard error, range, percentage). Each domain is scored between 0 and 1. In the modified NOS, scoring ranges from 0 to 5, with scores ≥3 indicating a low risk of bias and scores <3 denoting a high risk of bias. The quality evaluation was performed independently by two reviewers, and a third was involved in case of disagreement. Finally, articles that exhibited a low risk of bias were selected for the analyses (Additional file 2). All studies reviewed in this research were low-risk. Inter-rater reliability of reviewers regarding study relevancy was high (Kappa = 0.88).

### Statistical analyses

To calculate the pooled prevalence of anxiety in infertile women, the Metan command in Stata (version 14) was run. The heterogeneity of the studies was determined using the I^2^ statistical index, which ranges from 0 to 100; the larger the index, the more heterogeneous the findings. The categories encompassed by the I^2^ index were defined by Higgins as low heterogeneity (25%), moderate heterogeneity (50%), and high heterogeneity (75%). A study heterogeneity > 50% prompts the use of random effects models [30]. To identify the origins of heterogeneity in the examined studies, meta-regression was used to inquire into the types of tools used to measure anxiety, the duration of infertility, and sample size. Sensitivity analysis was also conducted to determine the effect of each study on the final results; that is, a given study was excluded from the final analysis, and the results were compared with and without the inclusion of the aforementioned study. An Egger test was performed to examine publication bias, and a subgroup analysis was carried out on the basis of the World Bank’s classification of countries by income and used the instruments. Because of small number of include studies, the significance level of statistical tests was set at 0.10.

## Results

**Fig. 1:**
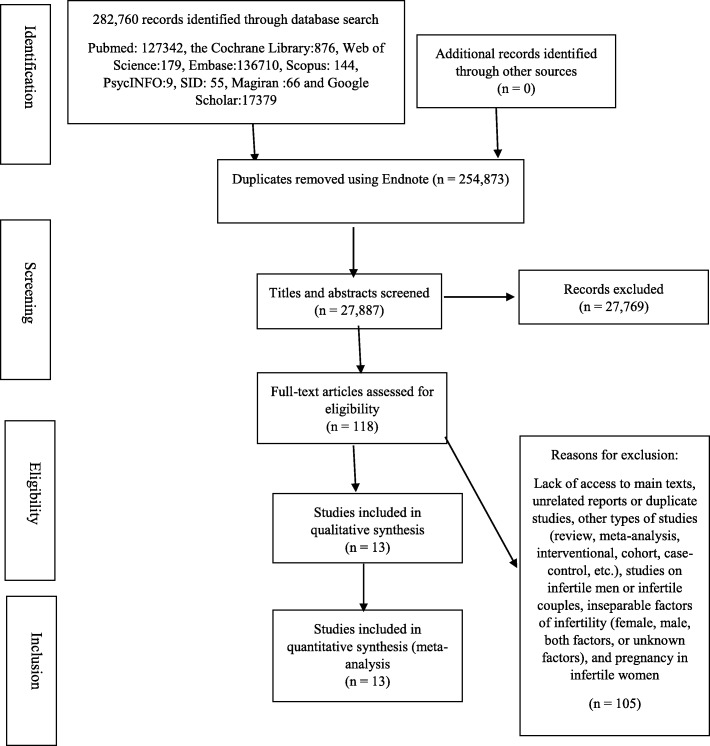
Flow diagram of the search for studies subjected to meta-analysis

Fig. 1 presents the flow diagram of the meta-analysis. As previously stated, an initial 282,760 articles were found using the keywords; the removal of duplicate studies yielded 27,887 works, which were screened on the basis of their titles and abstracts. A set of 118 relevant articles were obtained, after which a final sample of 13 cross-sectional studies were assessed in terms of quality after the exclusion of ineligible research. The quality assessment was performed independently by two reviewers (Fig. 1).

The selected studies encompassed one research each conducted in Nigeria, Hungary, and Finland; two investigations each carried out in Norway, Sweden, and Saudi Arabia; and four studies performed in Iran. Five studies were performed in the context of low- and middle-income countries, and eight were conducted in high-income countries. The following instruments were used in the evaluated works: The Primary Care Evaluation of Mental Disorders (one study), the Munich-Composite International Diagnostic Interview (one study), the Spielberger Trait Anxiety Inventory (one study), Zung’s Self-Rating Anxiety Scale (one study), the Mini International Neuropsychiatric Interview (two studies), the Cattle questionnaires (two studies), and the Hospital Anxiety and Depression Scale (five studies). The infertile women involved in the reviewed research numbered a total of 5055, and the sample size in each study ranged from 30 to 1413. The findings of the studies indicated that the maximum and minimum anxiety prevalence rates among the infertile women were 86.8 and 8.8%, respectively. Characteristics of the studies are shown in Table 1.

**Table 1:** Characteristics of the studies selected for the meta-analysis

ID	Authors	Years published	Countries	Income levels	Sample sizes	Ages (Y) (mean ± SD)	Mean years of infertility (mean ± SD)	Types of tools used	Quality assessment
1	Volgsten et al. [25]	2008	Sweden	High	413	32.9 ± 3.9	3.27 ± 1.63	PRIME-MD	5/5
2	Ramezanzadeh et al. [19]	2004	Iran	Low and middle	370	28 ± 5.37	6.36 ± 4.18	Cattle questionnaires	3/5
3	Klemetti et al. [31]	2010	Finland	High	239	37.4 ± 0.45	NA	M-CIDI	4/5
4	Alosaimi et al. [23]	2015	Saudi Arabia	High	206	NA	5.4 ± 4.3	MINI	4/5
5	Alshahrani et al. [32]	2019	Saudi Arabia	High	206	NA	NA	MINI	4/5
6	Maroufizadeh et al. [24]	2018	Iran	Low and middle	649	31/37 ± 5.69	5.62 ± 4.03	HADS	4/5
7	Joelsson et al. [33]	2017	Sweden	High	468	30.1 ± 4.8	1.8 ± 002	HADS	5/5
8	Lakatos et al. [34]	2017	Hungary	High	134	33.30 ± 4.85	3.61 ± 3.08	STAI-T	4/5
9	Biringer et al. [35]	2015	Norway	High	615	35.15 ± 6.28	NA	HADS	5/5
10	Upkong and Orgi [36]	2006	Nigeria	Low and middle	112	34.5 ± 5.5	4.46 ± 3.73	HADS	4/5
11	Rostad et al. [37]	2014	Norway	High	1413	NA	NA	HADS	5/5
12	Kalkhoran et al. [38]	2011	Iran	Low and middle	30	29.2 0 ± 4.2	4.8 ± 0.5	ZAS	3/5
13	Peyvandi et al. [39]	2009	Iran	Low and middle	200	33.39 ± 0.2	4.1 ± 0.6	Cattle questionnaires	4/5

*Abbreviations*: *PRIME-MD* Primary Care Evaluation of Mental Disorders, *NA* Not reported, *M-CIDI* Composite International Diagnostic Interview, Munich version, *MINI* Mini International Neuropsychiatric Interview, *HADS* Hospital Anxiety and Depression Scale, *STAI-T* Spielberger Trait Anxiety Inventory, *ZAS* Zung’s Self-Rating Anxiety Scale

### Evaluation of heterogeneity, publication bias and meta-regression

**Fig. 2:**
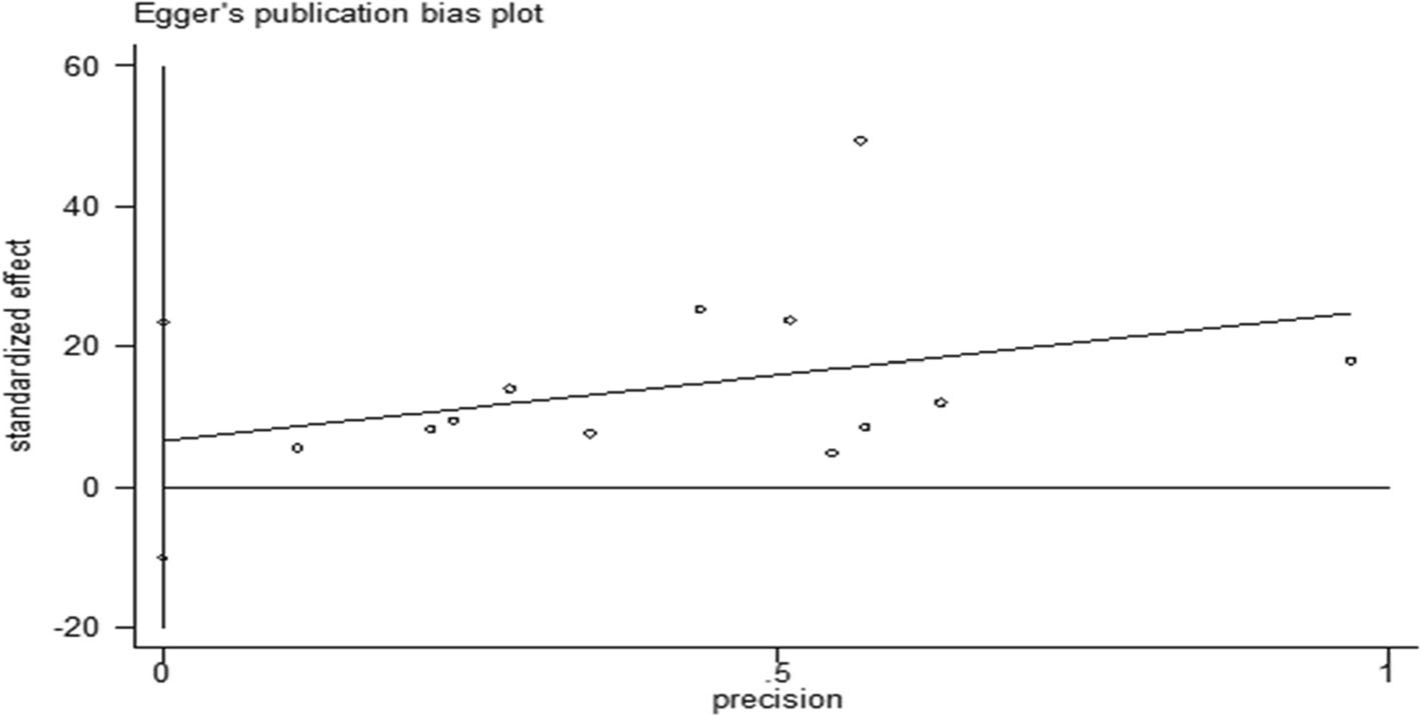
Plot of publication bias in relation to prevalence of anxiety

The I^2^ test results denoted heterogeneity in the studies (I^2^ = 99.3), thus compelling the use of a random effects model in the data analysis. The results of the Egger test indicated no evidence of publication bias (P _Egger’s Test_ = 0.406) (Fig. 2). To investigate the reasons for the heterogeneity in the assessed studies, three variables were introduced as covariance variables in the univariate meta-regression. The results revealed that sample size (*P* = 0.548), tool used (*P* = 0.691), and infertility duration (*P* = 0.554) were not accountable for the heterogeneity in the prevalence of anxiety in the infertile women.

### Meta-analysis

**Fig. 3:**
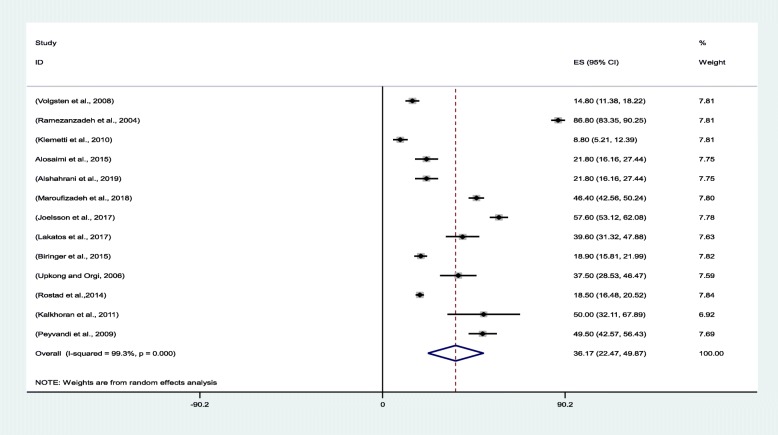
Forest plot for estimating the pooled prevalence of anxiety among infertile women

The pooled prevalence of anxiety in the evaluated studies was 36.17% [95% confidence interval (CI): 22.47–49.87]. Klemetti et al. [31]. and Ramezanzadeh et al. [19] reported the lowest (8.8%; 95% CI: 5.21–12.39) and highest (86.80%; 95% CI: 83.35–90.25) anxiety prevalence rates for Finland and Iran, respectively (Fig. 3).

### Subgroup analysis

**Fig. 4:**
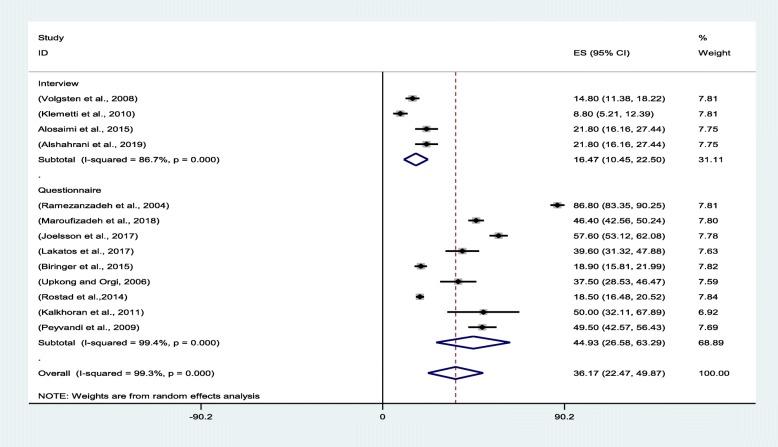
Forest plot of the pooled prevalence of anxiety based on instruments

The review of the literature revealed that the prevalence of anxiety among infertile women is greater in the studies that used a questionnaire to diagnose anxiety symptoms. Hence, the instruments were classified into two groups: one comprising clinical interview and another questionnaire. The findings reflected that the pooled anxiety prevalence levels were 16.47% (95% CI:10.45–22.50) and 44.93% (95% CI: 26.85–63.29) in clinical interview and questionnaire, respectively (Fig. 4).

**Fig. 5:**
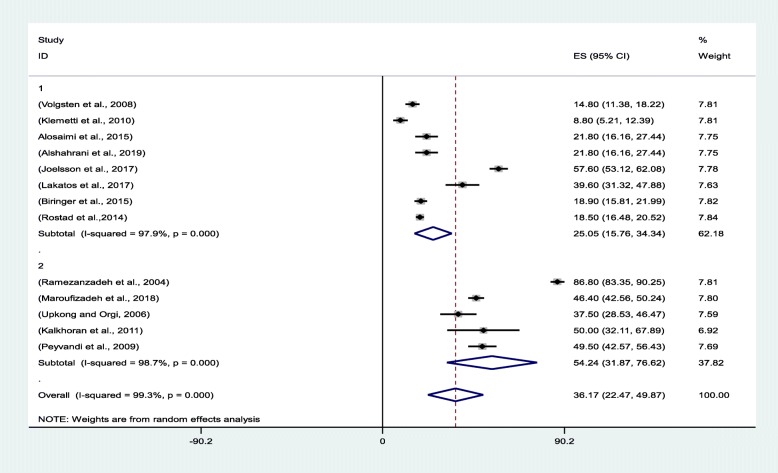
Forest plot of the pooled prevalence of anxiety based on country income

The review of the literature revealed that the prevalence of anxiety among infertile women is greater in low- and middle-income countries. Hence, the countries were classified into two groups: one comprising high-income nations and another consisting of low- and middle-income countries. The findings reflected that the pooled anxiety prevalence levels were 54.24% (95% CI: 31.86–78.62) and 25.05% (95% CI: 15.76–34.34) in low- and middle-income and high-income countries, respectively (Fig. 5).

## Discussion

This systematic and meta-analytic research evaluated prevalence of anxiety in infertile women on the basis of female factors. The results showed a prevalence rate of 36.17%, which is greater than the prevalence of the condition among the general public and healthy women. In a systematic review on anxiety prevalence in an adult population, the pooled prevalence of anxiety disorders was 3.8 to 25%%, 5.2 to 8.7% and 2.5 to 9.1%, respectively in adult population, women and young adults [40]. In research performed in an international context, the pooled one-year and lifetime prevalence rates of total anxiety disorders were estimated at 10.6% (95% CI: 7.5, 14.3%) and 16.6% (95% CI: 12.7, 21.1%), respectively [41].

Anxiety is a disturbing state that causes physical and mental stress in individuals over time [42]. A review of studies conducted in many countries suggested that women endure the major burdens of infertility and experience intense anxiety from being blamed for their failure to give birth [15]. Infertile women incur high costs from infertility treatments and suffer from problems caused by frequent visits to doctors, planned intercourse, and many other social issues, which strongly influence their mental health and anxiety levels [7]. The prevalence rate found in the current research demonstrated that anxiety prevalence was higher among women who experience difficulty conceiving than among females in pre-and post-natal stages. In their systematic review, Sawyer et al. [43] reported a 14.8% prevalence of anxiety in infertile women [95% CI: 12.3, 17.4%] and a 14% prevalence among women in pre- and post-natal periods [95% CI: 12.9, 15.2%]. In a meta-analysis conducted by Dennis et al. [44], the prevalence of self-reported anxiety symptoms was 18.2% (95% CI: 13.6–22.8) in the first trimester of pregnancy, 19.1% (95% CI: 15.9–22.4) in the second trimester of pregnancy, 24.6% (95% CI: 21.2–28.0) in the third trimester of pregnancy, and 15.0% (95% CI: 13.7–16.4) in the first to 14th weeks of the post-natal period.

In most societies, having a child is closely related to the identity of a woman, and being a mother is equated with being a female [11]. Under such perceptions, therefore, infertility brings women a sense of worthlessness, resulting in high levels of stress [45]. Because pregnancy is a critical goal in women’s lives, infertility causes a stronger sense of scarcity and anxiety in infertile women than their pregnant counterparts [46]. Females who are unable to conceive perceive their social security to be at risk and become anxious because they foresee a future with no child to take care of them in old age or in case of illness [47]. Note, however, that certain studies revealed no significant difference in anxiety levels between women undergoing reproductive health treatments and control groups, but such works inadequately elaborated on infertility-related factors (female and male determinants separately, both factors, unknown factors) [12, 48, 49].

The anxiety prevalence rate found in the present study (i.e., 36.17%) is also greater than the prevalence rate found among infertile men. In Peterson et al.’s [50] research on infertile men under treatment, for instance, 7% of the subjects reported experiencing anxiety. Alshahrani et al. [32] found an anxiety prevalence of 20.5% among infertile males. Regardless of whether they or their husbands are the cause of infertility, women suffer from greater pressure than men; because of social prejudices, infertility is more likely to be viewed as a female problem so that women face more family problems than do men [15] and are exposed to twice the risk of anxiety encountered by males [40] Furthermore, untreated anxiety in women is associated with frequent visits to physicians, the failure of infertility treatments, and reduced social relations—problems that point to the need for serious attention and intervention [51].

In the articles reviewed in the current meta-analysis, the prevalence of anxiety ranged from 8.8 to 86.8%, which reflect wide variations. Specific cases are those of Volgsten et al. [25] and Klemetti et al. [31], who reported prevalence levels of 8.8 and 14.8% in Sweden and Finland, respectively. Contrastingly, Upkong and Orgi [36] and Maroufizadeh et al. [24] found prevalence rates of 37.5 and 58.1% in Nigeria and Iran, respectively. The range also varied in the other studies as anxiety seemed more likely to prevail among infertile women in low-income countries. Hence, the subgroup analysis was based on the income of a given country in accordance with the World Bank’s classification.

The subgroup analysis showed that the prevalence of anxiety in infertile women in middle- and low-income countries was almost twice as much as that in high-income countries. Social and economic status, social support, the quality of marital relationships, child-rearing culture, and the availability of health facilities were among the factors influencing the prevalence of anxiety, with the low- and middle-income countries facing numerous problems in these respects [52, 53] Such situation exacerbates the burden of disease and disability in the aforementioned regions [54]. The annual spending of low-income countries on the treatment and prevention of mental diseases account for less than US$2 per person, whereas that in high-income countries have a budget of more than US$50 per year [55]. Women, especially infertile females, in middle - and low-income nations not only grapple with anxiety and mental illness but also contend with inadequate treatment facilities given that their mental problems are unrecognized in many cases [40]. In these countries, infertile women also experience high costs of infertility treatments and transportation to infertility centers, the lack of protective policies and regulations, and the lack of valuation from their families and communities [10]. Following anxiety occurrence and the lack of diagnosis and treatment, the failure of infertility treatments and defective cycles in intervention might emerge [56]. It is important to provide infertility policy based on the human rights framework [47]. In a 1994 international conference on population and development, the condition was highlighted as a global problem and a cause of substantial loss to reproductive health [57]. In the same vein, the WHO recommended the integration of infertility treatments into primary health services, especially in countries with low and medium income levels [1].

In a 2015 meta-analysis aimed at assessing the effectiveness of psychosocial interventions in infertile women and men, meta-regression findings revealed that decreased anxiety is associated with excellent pregnancy rates [42]. Anxiety is globally recognized as an important determinant of low health and involvement in the use of health services [58]. Organizing support groups for infertile women and providing psychological interventions that emphasize training on coping skills, cognitive behavioral therapy, and anxiety management can exert positive effects on anxiety control in infertile women and their treatment process [24].

The main limitations of the articles were their failure to distinguish between infertility-related factors and ascertain the mean values and standard deviations of anxiety measurement tools; these values could not be converted into prevalence rates. On the other hand, there is the diversity of instruments to determine the prevalence of anxious symptoms or anxiety in our study. This element, however, cannot be overlooked because the questionnaires provide quantitative information that can be analyzed and diagnosis cannot be made from these evaluations to the patients. Along with the questionnaire, clinical interviews should be considered for a more accurate diagnosis. Another shortcoming was the use of various instruments for assessing anxiety, in a general population. None of the tools was developed specifically to investigate incidence regarding female factors. Considering the various physical, psychological, and social differences among women and their exposure to infertility problems, the development of a specific questionnaire is essential. Generally, the definition of anxiety used is questionable because anxiety does not necessarily mean disturbance. In fact, it is a psychophysiological defense mechanism. The disorder appears when the intensity is extreme and frequent. There is no proportion between the stimulus that causes it and the symptomatology.

## Conclusion

The results of the systematic review and meta-analysis showed that the prevalence of anxiety symptoms in infertile women with female factors was 36.17%, which is greater than the rate found for the general population, pregnant women, and men. The effects that anxiety poses on quality of life, marital relations, therapeutic outcomes, and women’s authority in the family require serious consideration and intervention, especially in low- and middle-income countries. Despite extensive advances in this field, many issues remain unexplored, thus hindering the development of interventions intended to support infertile women and provide positive supportive measures to ensure positive outcomes during the therapeutic process.

## Supplementary information

**Additional file 1.** Search strategy

**Additional file 2.** Quality Assessment

## Data Availability

The datasets used and/or analyzed during the current study are available from the corresponding author on reasonable request.

## Abbreviations

WHO: World Health Organization
PRISMA: The preferred reporting items for systematic reviews and meta-analyses
NOS: Newcastle–Ottawa Scale
PRIME-MD: Primary Care Evaluation of Mental Disorders
NA: Not reported
M-CIDI: Composite International Diagnostic Interview, Munich version
MINI: Mini International Neuropsychiatric Interview
HADS: Hospital Anxiety and Depression Scale
STAI-T: Spielberger Trait Anxiety Inventory
ZAS: Zung’s Self-Rating Anxiety Scale
CI: Confidence Interval

## References

[1] Organization WH (2015). Global prevalence of infertility, infecundity and childlessness.

[2] Hanson B, Johnstone E, Dorais J, Silver B, Peterson CM, Hotaling J (2017). Female infertility, infertility-associated diagnoses, and comorbidities: a review. J Assist Reprod Genet.

[3] Mascarenhas MN, Flaxman SR, Boerma T, Vanderpoel S, Stevens GA (2012). National, regional, and global trends in infertility prevalence since 1990: a systematic analysis of 277 health surveys. PLoS Med.

[4] Fritz MA, Speroff L. Clinical gynecologic endocrinology and infertility. Philadelphia: Wolters Kluwer Health/Lippincott Williams & Wilkins; 2011.

[5] Ogawa M, Takamatsu K, Horiguchi F (2011). Evaluation of factors associated with the anxiety and depression of female infertility patients. BioPsychoSocial Med.

[6] Rooney KL, Domar AD (2018). The relationship between stress and infertility. Dialogues Clin Neurosci.

[7] Hasanpoor-Azghady SB, Simbar M, Vedadhir AA, Azin SA, Amiri-Farahani L (2019). The social construction of infertility among Iranian infertile women: a qualitative study. J Reprod Infertility.

[8] Albayrak E, Günay O (2007). State and trait anxiety levels of childless women in Kayseri, Turkey. Eur J Contracept Reprod Health Care.

[9] Vitale SG, La Rosa VL, Rapisarda AMC, Lagana AS (2017). Psychology of infertility and assisted reproductive treatment: the Italian situation. J Psychosom Obstet Gynecol.

[10] Morshed-Behbahani B, Lamyian M, Joulaei H, Montazeri A (2020). Analysis and exploration of infertility policies in Iran: a study protocol. Health Res Policy Syst.

[11] Kiani Z, Simbar M (2019). Infertility’s hidden and evident dimensions: a concern Requir-ing special attention in Iranian society. Iran J Public Health.

[12] Verhaak CM, Smeenk J, Evers A, Kremer JA, Kraaimaat F, Braat D (2006). Women’s emotional adjustment to IVF: a systematic review of 25 years of research. Hum Reprod Update.

[13] Monga M, Alexandrescu B, Katz SE, Stein M, Ganiats T (2004). Impact of infertility on quality of life, marital adjustment, and sexual function. Urology..

[14] Taebi M, Gandomani SJ, Nilforoushan P, GholamiDehaghi A (2016). Association between infertility factors and non-physical partner abuse in infertile couples. Iran J Nurs Midwifery Res.

[15] Bokaie M, Simbar M, Yassini-Ardekani S. Social factors affecting the sexual experiences of women faced with infertility: a qualitative study. Koomesh.2018;20(2):228-39.

[16] Onat G, Beji NK (2012). Effects of infertility on gender differences in marital relationship and quality of life: a case-control study of Turkish couples. Eur J Obstet Gynecol Reprod Biol.

[17] Drosdzol A, Skrzypulec V (2009). Evaluation of marital and sexual interactions of polish infertile couples. J Sex Med.

[18] Maroufizadeh S, Karimi E, Vesali S, Omani SR (2015). Anxiety and depression after failure of assisted reproductive treatment among patients experiencing infertility. Int J Gynecol Obstet.

[19] Ramezanzadeh F, Aghssa MM, Abedinia N, Zayeri F, Khanafshar N, Shariat M (2004). A survey of relationship between anxiety, depression and duration of infertility. BMC Womens Health.

[20] Hashemi S, Simbar M, Ramezani-Tehrani F, Shams J, Majd HA (2012). Anxiety and success of in vitro fertilization. Eur J Obstet Gynecol Reprod Biol.

[21] El Kissi Y, Romdhane AB, Hidar S, Bannour S, Idrissi KA, Khairi H (2013). General psychopathology, anxiety, depression and self-esteem in couples undergoing infertility treatment: a comparative study between men and women. Eur J Obstet Gynecol Reprod Biol.

[22] Zivaridelavar M, Kazemi A, Kheirabadi GR. The effect of assisted reproduction treatment on mental health in fertile women. J Educ Health Promot. 2016:5. 10.4103/2277-9531.184552. 10.4103/2277-9531.184552 PMC4959257 27512701

[23] Alosaimi FD, Altuwirqi MH, Bukhari M, Abotalib Z, BinSaleh S (2015). Psychiatric disorders among infertile men and women attending three infertility clinics in Riyadh, Saudi Arabia. Ann Saudi Med.

[24] Maroufizadeh S, Ghaheri A, Almasi-Hashiani A, Mohammadi M, Navid B, Ezabadi Z (2018). The prevalence of anxiety and depression among people with infertility referring to Royan Institute in Tehran, Iran: a cross-sectional questionnaire study. Middle East Fertil Soc J.

[25] Volgsten H, Skoog Svanberg A, Ekselius L, Lundkvist Ö, Sundström PI (2008). Prevalence of psychiatric disorders in infertile women and men undergoing in vitro fertilization treatment. Hum Reprod.

[26] Haidich A-B (2010). Meta-analysis in medical research. Hippokratia.

[27] Norman G, Faria R, Paton F, Llewellyn A, Fox D, Palmer S (2013). The preferred reporting items for systematic reviews and meta-analyses. Omalizumab for the treatment of severe persistent allergic asthma: a systematic review and economic evaluation: NIHR Journals Library.

[28] Stang A (2010). Critical evaluation of the Newcastle-Ottawa scale for the assessment of the quality of nonrandomized studies in meta-analyses. Eur J Epidemiol.

[29] Zhang L, Fu T, Yin R, Zhang Q, Shen B (2017). Prevalence of depression and anxiety in systemic lupus erythematosus: a systematic review and meta-analysis. BMC Psychiat.

[30] Higgins J, Thompson S (2002). Quantifying heterogeneity in a meta-analysis. Stat Med.

[31] Klemetti R, Raitanen J, Sihvo S, Saarni S, Koponen P (2010). Infertility, mental disorders and well-being–a nationwide survey. Acta Obstet Gynecol Scand.

[32] Alshahrani SS, Tashkandi AM, Alolayah AM (2019). Relationship between infertile men and women with psychiatric disorder in Saudi Arabia. Indo Am J Pharm Sci.

[33] Joelsson LS, Tydén T, Wanggren K, Georgakis M, Stern J, Berglund A (2017). Anxiety and depression symptoms among sub-fertile women, women pregnant after infertility treatment, and naturally pregnant women. Eur Psychiat.

[34] Lakatos E, Szigeti JF, Ujma PP, Sexty R, Balog P (2017). Anxiety and depression among infertile women: a cross-sectional survey from Hungary. BMC Womens Health.

[35] Biringer E, Howard LM, Kessler U, Stewart R, Mykletun A (2015). Is infertility really associated with higher levels of mental distress in the female population? Results from the north-Trøndelag health study and the medical birth registry of Norway. J Psychosom Obstet Gynecol.

[36] Upkong D, Orgi E (2006). Mental health of infertile women in Nigeria. Turk J Psychiatry.

[37] Rostad B, Schmidt L, Sundby J, Schei B (2014). Infertility experience and health differentials–a population-based comparative study on infertile and non-infertile women (the HUNT study). Acta Obstet Gynecol Scand.

[38] Kalkhoran LF, Bahrami H, Farrokhi NA, Zeraati H, Tarahomi M. Comparing anxiety, depression and sexual life satisfaction in two groups of fertile and infertile women in Tehran. J Reprod Infertility. 2011;12(2):157-62.

[39] Peyvandi S, Hosseini H, Daneshpour M, Mohammadpour R, Golami N (2009). Frequency of depression, anxiety and marital satisfaction Related Factors in Infertile Women Referring to Centers Infertility in Sari city. J Mazandaran Univ Med Sci.

[40] Remes O, Brayne C, Van Der Linde R, Lafortune L (2016). A systematic review of reviews on the prevalence of anxiety disorders in adult populations. Brain Behav.

[41] Somers JM, Goldner EM, Waraich P, Hsu L (2006). Prevalence and incidence studies of anxiety disorders: a systematic review of the literature. Can J Psychiatry.

[42] Frederiksen Y, Farver-Vestergaard I, Skovgård NG, Ingerslev HJ, Zachariae R (2015). Efficacy of psychosocial interventions for psychological and pregnancy outcomes in infertile women and men: a systematic review and meta-analysis. BMJ Open.

[43] Sawyer A, Ayers S, Smith H (2010). Pre-and postnatal psychological wellbeing in Africa: a systematic review. J Affect Disord.

[44] Dennis C-L, Falah-Hassani K, Shiri R (2017). Prevalence of antenatal and postnatal anxiety: systematic review and meta-analysis. Br J Psychiatry.

[45] Batool SS, de Visser RO (2014). Psychosocial and contextual determinants of health among infertile women: a cross-cultural study. Psychol Health Med.

[46] Matsubayashi H, Hosaka T, Izumi S-i, Suzuki T, Makino T (2001). Emotional distress of infertile women in Japan. Hum Reprod.

[47] Gerrits T, Van Rooij F, Esho T, Ndegwa W, Goossens J, Bilajbegovic A (2017). Infertility in the Global South: Raising awareness and generating insights for policy and practice. Facts Views Vision ObGyn.

[48] Schmidt L (2009). Social and psychological consequences of infertility and assisted reproduction–what are the research priorities?. Hum Fertil.

[49] Milazzo A, Mnatzaganian G, Elshaug AG, Hemphill SA, Hiller JE, Group AHS (2016). Depression and anxiety outcomes associated with failed assisted reproductive technologies: a systematic review and meta-analysis. PLoS One.

[50] Peterson BD, Newton CR, Feingold T (2007). Anxiety and sexual stress in men and women undergoing infertility treatment. Fertil Steril.

[51] Karimzadeh M, Salsabili N, Asbagh FA, Teymouri R, Pourmand G, Naeini TS (2017). Psychological disorders among Iranian infertile couples undergoing assisted reproductive technology (ART). Iran J Public Health.

[52] Fisher J (2012). Mello MCd, Patel V, Rahman a, Tran T, Holton S, et al. prevalence and determinants of common perinatal mental disorders in women in low-and lower-middle-income countries: a systematic review. Bull World Health Organ.

[53] Layte R (2011). The association between income inequality and mental health: testing status anxiety, social capital, and neo-materialist explanations. Eur Sociol Rev.

[54] Lund C, Breen A, Flisher AJ, Kakuma R, Corrigall J, Joska JA (2010). Poverty and common mental disorders in low and middle income countries: a systematic review. Soc Sci Med.

[55] Wang PS, Aguilar-Gaxiola S, Alonso J, Angermeyer MC, Borges G, Bromet EJ (2007). Use of mental health services for anxiety, mood, and substance disorders in 17 countries in the WHO world mental health surveys. Lancet.

[56] Gerrits T (2012). Biomedical infertility care in low resource countries: barriers and access. Facts Views Vis Obgyn Monog.

[57] Fincher RA (1994). International Conference on Population and Development. Envtl Pol’y & L.

[58] Simpson HB, Neria Y, Lewis-Fernández R, Schneier F (2010). Anxiety disorders: theory, research and clinical perspectives: Cambridge University Press.

