# Supplementary material for: The prevalence of anxiety symptoms in infertile women: a systematic review and meta-analysis
Source: Fertil Res Pract. 2020 Apr 15;6:7. doi: 10.1186/s40738-020-00076-1 (PMC7157980; doi:10.1186/s40738-020-00076-1)
Supplement: Supplementary file 1 — Additional file 1. Search strategy [file 40738_2020_76_MOESM1_ESM.doc]

**Appendix 1: Search strategy**

(Anxiety[Title/Abstract]) OR Hypervigilance[Title/Abstract]) OR Nervousness[Title/Abstract]) OR Social Anxiety[Title/Abstract]) OR Social Anxieties[Title/Abstract]) OR Anxiety Disorders[Title/Abstract]) OR Anxiety Disorder[Title/Abstract]) OR Anxiety Neuroses[Title/Abstract]) OR Neurotic Anxiety State[Title/Abstract]) OR Neurotic Anxiety States[Title/Abstract]) AND infertility[Title/Abstract]) OR Sterility[Title/Abstract]) OR Reproductive Sterility[Title/Abstract]) OR Subfertility[Title/Abstract]) OR Sub-Fertility[Title/Abstract]) AND Prevalence[Title/Abstract]) OR Epidemiology[Title/Abstract]).
