# Supplementary material for: The prevalence of anxiety symptoms in infertile women: a systematic review and meta-analysis
Source: Fertil Res Pract. 2020 Apr 15;6:7. doi: 10.1186/s40738-020-00076-1 (PMC7157980; doi:10.1186/s40738-020-00076-1)
Supplement: Supplementary file 2 — Additional file 2. Quality Assessment [file 40738_2020_76_MOESM2_ESM.doc]

**Appendix 2**: Quality Assessment

Modified Newcastle-Ottawa scoring guide.

**(1) Representativeness of the sample:**

1 point: Population contained a mixture of specialties at multiple sites.

0 points: Population contained a single specialty at a single site.

**(2) Sample size:**

1 point: Sample size was 200 and greater than 200 participants.

0 points: Sample size was less than 200 participants or a convenience sample.

**(3) Non-respondents:**

1 point: Comparability between respondent and non-respondent characteristics was established, and the response rate was satisfactory.

0 points: The response rate was unsatisfactory, the comparability between respondents and non-respondents was unsatisfactory, or there was no description of the response rate or the characteristics of the responders and the non-responders.

**(4) Ascertainment of anxiety:**

1 point: Validated measurement tool using a validated cutoff score or clinical interview.

0 points: Non-validated measurement tool, or validated measurement tool with non-valid cutoff score, or 2-item PRIME-MD (scored as such due to its low specificity).

**(5) Quality of descriptive statistics reporting:**

1 point: Reported descriptive statistics to describe the population (*e.g.*, age, sex) with proper measures of anxiety (*e.g.*, standard deviation, standard error, range, precentage).

0 points: Descriptive statistics were not reported, were incomplete, or did not include proper measures of anxiety.

**Legend:** This scale, the scoring of which ranges from 0 to 5, assesses quality in several domains: sample representativeness and size, comparability between respondents and non-respondents, ascertainment of depressive symptoms, and statistical quality. Studies were judged to be of low risk of bias (≥3 points) or high risk of bias (<3 points).

**Total = /5**

|  | **Results of Newcastle-Ottawa Risk of Bias Assessment** | | | | | | |
| --- | --- | --- | --- | --- | --- | --- | --- |
|  | **Study ID** | Representativeness | Size | Comparability | Outcome | Statistics | Total |
| **1** |  | 1 | 1 | 1 | 1 | 1 | 5/5 |
| **2** |  | 0 | 1 | 0 | 1 | 1 | 3/5 |
| **3** |  | 1 | 1 | 0 | 1 | 1 | 4/5 |
| **4** |  | 1 | 1 | 0 | 1 | 1 | 4/5 |
| **5** |  | 1 | 1 | 0 | 1 | 1 | 4/5 |
| **6** |  | 0 | 1 | 1 | 1 | 1 | 4/5 |
| **7** |  | 1 | 1 | 1 | 1 | 1 | 5/5 |
| **8** |  | 1 | 0 | 1 | 1 | 1 | 4/5 |
| **9** |  | 1 | 1 | 1 | 1 | 1 | 5/5 |
| **10** |  | 1 | 0 | 1 | 1 | 1 | 4/5 |
| **11** | **(Rostad et al., 2014)** | 1 | 1 | 1 | 1 | 1 | 5/5 |
| **12** |  | 0 | 0 | 1 | 1 | 1 | 3/5 |
| **13** |  | 1 | 1 | 0 | 1 | 1 | 4/5 |
